# Predictions of Conjugate Heat Transfer in Turbulent Channel Flow Using Advanced Wall-Modeled Large Eddy Simulation Techniques

**DOI:** 10.3390/e23060725

**Published:** 2021-06-07

**Authors:** Yongxiang Li, Florian Ries, Kaushal Nishad, Amsini Sadiki

**Affiliations:** 1 Department of Mechanical Engineering, Institute of Reactive Flows and Diagnostics, Technical University of Darmstadt, Otto-Berndt-Str. 3, 64287 Darmstadt, Germany; ries@ekt.tu-darmstadt.de (F.R.); nishad@ekt.tu-darmstadt.de (K.N.); sadiki@ekt.tu-darmstadt.de (A.S.); 2 Department of Mechanical Engineering, Institute of Energy and Power Plant Technology, Technical University of Darmstadt, Otto-Berndt-Str. 3, 64287 Darmstadt, Germany; 3 Laboratoire de Genies des Procedes et Thermodynamique, Institut Superieur des Sciences et Techniques Appliquees, B.P. 6534 Kinshasa, Democratic Republic of the Congo

**Keywords:** turbulent flows, conjugate heat transfer, large eddy simulation, near-wall modeling, wall functions, zonal RANS–LES, improved delayed detached eddy simulation, turbulent heated channel flow, entropy generation analysis

## Abstract

In this paper, advanced wall-modeled large eddy simulation (LES) techniques are used to predict conjugate heat transfer processes in turbulent channel flow. Thereby, the thermal energy transfer process involves an interaction of conduction within a solid body and convection from the solid surface by fluid motion. The approaches comprise a two-layer RANS–LES approach (zonal LES), a hybrid RANS–LES representative, the so-called improved delayed detached eddy simulation method (IDDES) and a non-equilibrium wall function model (WFLES), respectively. The results obtained are evaluated in comparison with direct numerical simulation (DNS) data and wall-resolved LES including thermal cases of large Reynolds numbers where DNS data are not available in the literature. It turns out that zonal LES, IDDES and WFLES are able to predict heat and fluid flow statistics along with wall shear stresses and Nusselt numbers accurately and that are physically consistent. Furthermore, it is found that IDDES, WFLES and zonal LES exhibit significantly lower computational costs than wall-resolved LES. Since IDDES and especially zonal LES require considerable extra work to generate numerical grids, this study indicates in particular that WFLES offers a promising near-wall modeling strategy for LES of conjugated heat transfer problems. Finally, an entropy generation analysis using the various models showed that the viscous entropy production is zero inside the solid region, peaks at the solid–fluid interface and decreases rapidly with increasing wall distance within the fluid region. Except inside the solid region, where steep temperature gradients lead to high (thermal) entropy generation rates, a similar behavior is monitored for the entropy generation by heat transfer process.

## 1. Introduction

As classified in [1], heat transfer processes can be investigated using conjugate, coupled or adjoint formulations corresponding to problems that contain two or more subdomains with phenomena that are described by different differential equations. In many energy systems, such as internal combustion engines, gas turbines, heat exchangers and many more, like converter monolith channels in after-treatment devices, conjugate heat transfer features a thermal energy transfer process that involves the interaction of conduction within a solid body and convection from the solid surface by fluid motion [2]. Note that catalytic converters, which are usually multiple-channel reactors with a honeycomb structure, are additionally characterized by relatively low pressure drop, enhanced mass transfer and large geometric surface area and thickness of the catalyst film on the substrate wall in which heterogeneous chemical reactions take place [3]. The structural field of a solid wall in terms of structural stresses and deformation as well as chemical reactions is not considered in the present paper. Nevertheless, a realistic prediction of conjugate heat transfer problems is very challenging as it requires a coupling of the conduction in the solid part and the convection in the fluid region.

Focusing on the numerical simulation of turbulent conjugate heat transfer, the large eddy simulation (LES) technique has been proven to be an accurate approach to predict such turbulent thermal processes. This was shown in the literature for both generic flow configurations like heated cavity flows [4] and also for complex engineering applications such as cooling of gas turbine blades [5]. However, it is well established that the computational cost of LES with conjugated heat transfer is very high. This is mainly because of the thin momentum and thermal boundary layers at the solid surface that have to be fully resolved in classical LES which requires very fine spatial resolution, in particular for turbulent flows with high Reynolds and Prandtl numbers. Therefore, in order to overcome this issue, it is common practice in LES to use a near-wall modeling approach to reduce the required computational effort of the simulation. In general, such near-wall modeling strategies can be divided in the context of LES into approaches based on wall functions (WFLES), two-layer RANS–LES (zonal LES) and hybrid RANS–LES methods [6].

Regarding WFLES, the momentum and thermal boundary layers are not explicitly resolved by the numerical grid. Instead, they are bridged with a single cell while suitable assumptions are made about the near-wall velocity and temperature profiles [7]. Thereby, in the case of classical wall functions, a linear variation of the near-wall velocity is assumed very close to the wall and a semi-logarithmic variation away from it (e.g., [8,9,10]). Based on Reynolds analogy assumptions, similar variation is also found for the near-wall temperature profile (e.g., [11,12,13,14]). However, it is well known that such simplified formulations for the near-wall velocity and temperature do not apply to complex boundary layers because they are based on simple equilibrium flow assumptions. Therefore, advanced wall functions for the velocity (e.g., [7,15,16,17,18]) and also for the temperature [7,18] were proposed in the literature that account for additional non-equilibrium effects like time rate change, convection or pressure gradients. This allows accurate predictions of realistic heat and fluid flow applications with a reasonable computational cost, as was recently shown by the authors in [7].

In the case of the zonal LES approach, a numerical grid with a fine spatial resolution is embedded between the matching location of the outer mesh and the solid surface within the fluid region. A simplified set of RANS-based turbulent boundary-layer equations are solved at the embedded mesh region. By means of this, the required wall shear stress is calculated and employed as a wall boundary condition for the LES calculation on the overlapped outer mesh [6,19]. Thereby, pressure gradient and convection effects are taken into account from the solution of the RANS-based turbulent boundary-layer equations. Consequently, two-layer models are also able to capture, to some extent, non-equilibrium heat and fluid flow effects. Nevertheless, it was observed in many numerical studies [20,21,22] that two-layer models tend to overpredict the wall shear stress. In addition to this, the generation of two separate numerical grids can be very challenging, in particular for complex geometries, which impedes the use of zonal LES for practical engineering applications with conjugate heat transfer.

In hybrid RANS–LES modeling approaches, a RANS model is applied in the vicinity of the solid surface, while LES equations with a subgrid-scale model are solved away from it. In this framework, different strategies can be used for the transition from a RANS behavior to a LES behavior, based on criteria updated during the computation [23]. For instance, the turbulent length scale can be changed from a RANS mixing length scale to a grid size-related length scale, or a blending function can be used to merge the RANS and subgrid-scale eddy viscosities [6]. In contrast to wall-resolved LES of conjugate heat transfer, where the grid has to be refined isotropically in all three directions in the vicinity of the solid surface in the fluid region, hybrid RANS–LES requires only grid refinement in the wall-normal direction, leading to a significant reduction in the computational cost [24]. Prominent examples of hybrid RANS–LES models are detached eddy simulations (DESs) [25], delayed detached eddy simulations (DDESs) [26], improved delayed detached eddy simulations (IDDESs) [27], very large eddy simulations (VLESs) [28] or scale-adaptive simulations (SASs) [29].

From this short literature review, it appears that numerous LES near-wall modeling approaches have been proposed in the literature. However, it is worth mentioning that an assessment of the prediction accuracy and computational cost of these wall models regarding turbulent flow with conjugate heat transfer are rarely reported. This motivates the present work that reports on comparative predictions of conjugate heat transfer achieved by means of advanced near-wall modeling approaches in the context of LES for turbulent flows with conjugate heat transfer. For this purpose, wall-modeled LES of turbulent channel flow with conjugate heat transfer was conducted and near-wall statistics were compared with DNS and wall-resolved LES results [30]. A two-layer RANS–LES approach based on the Spalart–Allmaras model [31], an improved delayed detached eddy simulation method (IDDES) [27] and a non-equilibrium wall function model (WFLES) [7] were assessed in terms of prediction accuracy, physical consistency and computational cost. To the authors’ knowledge, this is the first study in the literature that presents (i) a comprehensive comparison study of zonal LES, IDDES and WFLES approaches in terms of conjugated heat transfer, (ii) a non-equilibrium WFLES approach applied to simulate conjugate heat transfer and (iii) predictions of entropy production rates based on wall-modeled LES in turbulent conjugate heated channel flow. Furthermore, wall-resolved LES data of conjugate heated channel flow at $Re_{\tau }=640$ are provided for evaluation purposes that to date have not been available in the literature.

This paper is organized as follows. The different near-wall modeling approaches are introduced in Section 2. Subsequently, the turbulent channel flow configuration with conjugate heat transfer is described in Section 3 and the numerical procedure employed for the simulation is outlined. Then, in Section 4, results of the wall-modeled LES are analyzed and the approaches are subsequently assessed. Afterward, an entropy generation analysis using the various models employed is reported. Finally, some concluding remarks are summarized in Section 5.

## 2. Wall-Modeled LES Approaches with Conjugate Heat Transfer

Since the structural field of the solid wall in terms of structural stresses and deformation and chemical reactions are not considered, the conjugate heat transfer problem under investigation in the present paper can be divided into three main regions, namely a non-isothermal fluid flow region, a transient heat conduction through the solid and a thermal solid/fluid interface. In the case of turbulent incompressible Navier–Stokes–Fourier fluid flow with conjugate heat transfer and constant physical properties, the transport equations for mass, momentum and energy with respect to RANS and LES can be formulated for the fluid region as:

(1) $$\frac{\partial {\bar{U}}_{i}}{\partial x_{i}}=0,$$

(2) $$\frac{\partial {\bar{U}}_{i}}{\partial t}+\frac{\partial }{\partial x_{j}}\left({\bar{U}}_{i}{\bar{U}}_{j}\right)=-\frac{\partial \bar{p}}{\partial x_{i}}+\frac{\partial }{\partial x_{j}}\left(\left(\nu +\nu_{t}\right)\left(\frac{\partial {\bar{U}}_{i}}{\partial x_{j}}+\frac{\partial {\bar{U}}_{j}}{\partial x_{i}}\right)\right)+{\bar{f}}_{i}^{U},$$

(3) $$\frac{\partial {\bar{T}}^{f}}{\partial t}+\frac{\partial }{\partial x_{i}}\left({\bar{U}}_{i}{\bar{T}}^{f}\right)=\frac{\partial }{\partial x_{i}}\left(\left(\alpha^{f}+\frac{\nu_{t}}{Pr_{t}}\right)\frac{\partial {\bar{T}}^{f}}{\partial x_{i}}\right)+{\bar{f}}^{T_{f}},$$

where the concept of eddy viscosity is applied in order to close the LES and RANS equations. In Equations (1)–(3), $U_{i}$ denotes the flow velocity, $T^{f}$ the fluid temperature, *p* the kinematic pressure, ν the kinematic viscosity, α the molecular thermal diffusivity and $f^{T_{f}}$, $f_{i}^{U}$ are additional source terms. Note that, in the present study, purely forced convection is investigated. Therefore, no additional source terms for buoyancy effects appear in the balance equations and the temperature is treated as a passive scalar.

Regarding LES, the operator $\bar{\left(•\right)}$ in Equations (1)–(3) represents spatially filtered quantities, $\nu_{t}$ is the subgrid-scale viscosity and $Pr_{t}$ the subgrid-scale Prandtl number. Thereby, the wall-adapting local eddy viscosity (WALE) model [7,19,32] is employed in this study to calculate $\nu_{t}$ and the subgrid-scale Prandtl number is set to $Pr_{t}=0.5$ in accordance with [30,33]. In the context of RANS, the operator $\bar{\left(•\right)}$ denotes time-averaged quantities, $\nu_{t}$ is the turbulent eddy viscosity and $Pr_{t}$ the turbulent Prandtl number. In this work, the Spalart–Allmaras turbulence model [31] is used to close the RANS equations and the turbulent Prandtl number is set to $Pr_{t}=1$. This turbulent Prandtl number is selected in accordance with the DNS study of turbulent heated channel flow of Kawamura et al. [34].

In the solid region, the velocity is zero in all the balance Equations (1)–(3) and only the energy equation has to be solved, which further simplifies to the classical heat equation:

(4) $$\frac{\partial T^{s}}{\partial t}=\frac{\partial }{\partial x_{i}}\left(\alpha^{s}\frac{\partial T^{s}}{\partial x_{i}}\right).$$

Here, $T^{s}$ represents the solid temperature and $\alpha^{s}$ the thermal diffusivity of the solid.

Finally, the solid and fluid regions are coupled via a thermal interface. Here, the temperature and the heat flux of both phases have to be equal, which leads to the following boundary conditions at the fluid–solid interface:

(5) $$T^{s}=T^{f}\;\rho^{f}c_{p}^{f}\left(\alpha^{f}+\alpha_{t}\right)\frac{dT^{f}}{dn}=\lambda^{s}\frac{dT^{s}}{dn},$$

where $\rho^{f}$ is the fluid density, $c_{p}^{f}$ the specific heat capacity of the fluid, $\lambda^{s}$ the thermal conductivity of the solid, $\alpha_{t}$ the turbulent thermal diffusivity and *n* represents the direction normal to the solid surface.

Next, the different LES near-wall modeling approaches applied in this study to model the momentum and thermal boundary layers in the vicinity of the solid surface are described.

### 2.1. LES with Non-Equilibrium Wall Functions (WFLES)

The basic idea of wall function models is to bridge the momentum and/or thermal boundary layers with a single grid cell and make suitable assumptions about the near-wall velocity and temperature profiles in order to obtain the required wall shear stress and wall heat flux, respectively. In this work, the non-equilibrium wall function approach as proposed by the authors in [7] is employed. In contrast to classical formulations, the non-equilibrium wall functions are continuously valid over the whole range of dimensionless wall distance $y^{+}$ and include transient as well as local non-equilibrium effects like time rate change, adverse pressure gradients, convection and additional source terms. In the following section, only the formulation of the non-equilibrium wall function for the momentum boundary layer is described. A similar procedure applies for the thermal boundary layer and is therefore not shown here for the sake of clarity. A detailed description as well as computational details on the non-equilibrium wall function approach can be found in [7].

Based on the momentum boundary layer equation, an analytical solution for the near-wall velocity profile can be formulated as [7]:

(6) $$\begin{array}{cc} U^{+} & =\frac{C_{U}^{+}}{\kappa }y^{+}+\left(\frac{\tau_{w}}{\rho u_{\tau }^{2}}-\frac{C_{U}^{+}}{\kappa }\right)U_{I}^{+}+\frac{\kappa C_{U}^{+}}{3a_{U}\kappa C_{0}-2C_{0}}\left(U_{II}^{+}+3\sqrt{\frac{a_{U}\kappa -1}{3a_{U}\kappa +1}}U_{III}^{+}\right),\\ U_{I}^{+} & =\frac{1}{\kappa }\log\left(\frac{y^{+}+a_{U}}{a_{U}}\right)-\frac{R^{2}}{a_{U}^{2}+4\gamma a_{U}}\left(\left(4\gamma -a_{U}\right)U_{II}^{+}+\frac{\gamma }{\beta }\left(4\gamma -5a_{U}\right)U_{III}^{+}\right),\\ U_{II}^{+} & =\log\left(\frac{a_{U}}{R}\frac{\sqrt{{\left(y^{+}-\gamma \right)}^{2}+\beta^{2}}}{y^{+}+a_{U}}\right),\\ U_{III}^{+} & =\arctan\left(\frac{y^{+}}{\beta }-\frac{\gamma }{\beta }\right)+\arctan\left(\frac{\gamma }{\beta }\right), \end{array}$$

with

(7) $$a_{U}=\Omega_{U}/\left(6\kappa {C_{0}}^{1/3}\right)+2{C_{0}}^{1/3}/\left(3\kappa \Omega_{U}\right)+\frac{1}{3k},$$

(8) $$\gamma =\left(-1/\kappa +a_{U}\right)/2,\;\beta =\sqrt{2a_{U}\gamma -\gamma^{2}},\;R=\sqrt{\gamma^{2}+\beta^{2}},$$

(9) $$\Omega_{U}=\left(108\kappa^{3}+8C_{0}\right.+{\left.12\sqrt{81k^{6}+12C_{0}\kappa^{3}}\right)}^{1/3},$$

where κ≈0.41 and $C_{0}=9.6\cdot 10^{-4}$. Here, $U^{+}=U/u_{\tau }$ and $y^{+}=yu_{\tau }/\nu$ are the dimensionless mean velocity and wall distance, respectively, and $u_{\tau }$ is the friction velocity. In this formulation, all local non-equilibrium effects are combined into $C_{U}^{+}=\nu C_{u}/\rho^{f}u_{\tau }^{3}$, where $C_{U}$ is calculated at the cell centroid *P* of the first cell at the wall as:

(10) $$C_{U}={\left[\frac{1}{2}\left(\frac{\partial \rho^{f}U_{\zeta }}{\partial t}+\frac{\partial }{\partial \zeta }\left(\rho^{f}U_{\zeta }U_{\zeta }\right)+\frac{\partial }{\partial \eta }\left(\rho U_{\eta }U_{\zeta }\right)\right)+\frac{\partial \rho^{f}p}{\partial \zeta }+\rho^{f}f_{\zeta }^{U}\right]}_{P},$$

with ζ as the flow direction and η the wall-normal direction.

Based on Equation (6), the required wall shear stress can be determined using an iterative procedure (e.g., Newton–Raphson or regula falsi methods) and applied as a boundary condition for the LES with the wall function approach. A similar procedure was used in this work to bridge the thermal boundary layer. Thereby, the thermal diffusivity $\alpha_{t}$ was calculated in an iterative procedure from the temperature wall function and employed as a boundary condition.

### 2.2. Two-Layer RANS–LES Approach (Zonal LES)

As mentioned above, in the two-layer RANS–LES approach (zonal LES), a numerical grid with a fine spatial resolution, is embedded between the matching location of the outer mesh and the solid surface within the fluid region. Thereby, in the present study, the Spalart–Allmaras [31] model was applied to solve the RANS equations at the inner layer on the embedded mesh. The Spalart–Allmaras RANS model reads [31]:

(11) $$\frac{\partial \tilde{\nu }}{\partial t}+\frac{\partial }{\partial x_{i}}\left({\bar{U}}_{i}\tilde{\nu }\right)=C_{b1}\tilde{S}\tilde{\nu }+\frac{1}{\sigma_{\nu_{t}}}\left[\frac{\partial }{\partial x_{j}}\left(\left(\nu +\tilde{\nu }\right)\frac{\partial \tilde{\nu }}{\partial x_{j}}\right)+C_{b2}\frac{\partial \tilde{\nu }}{\partial x_{j}}\frac{\partial \tilde{\nu }}{\partial x_{j}}\right]-C_{w1}f_{w}{\left(\frac{\tilde{\nu }}{\tilde{d}}\right)}^{2},$$

where the turbulent viscosity is calculated as

(12) $$\nu_{t}=\tilde{\nu }f_{\nu 1}\;f_{\nu 1}=\frac{\chi^{3}}{\chi^{3}+C_{\nu 1}^{3}},\;\chi :=\frac{\tilde{\nu }}{\nu }.$$

The coefficients in the Spalart–Allmaras RANS model are defined as

(13) $$\begin{array}{c} \tilde{S}=\sqrt{2{\bar{\Omega }}_{ij}{\bar{\Omega }}_{ij}}+\frac{\tilde{\nu }}{\kappa^{2}{\tilde{d}}^{2}}f_{\nu 2},\;f_{\nu 2}=1-\frac{\chi }{1+\chi f_{\nu 1}},\\ f_{w}=g{\left[\frac{1+C_{\omega 3}^{6}}{g^{6}+C_{\omega 3}^{6}}\right]}^{1/6},\;g=r+C_{\omega 2}\left(r^{6}-r\right),\;r:=\frac{\tilde{\nu }}{\tilde{S}\kappa^{2}{\tilde{d}}^{2}}, \end{array}$$

where ${\bar{\Omega }}_{ij}=1/2\left(\partial {\bar{U}}_{i}/\partial x_{j}-\partial {\bar{U}}_{j}/\partial x_{i}\right)$ is the rotation tensor and $\tilde{d}$ a characteristic length scale defined as the distance to the wall. The model constants are given as

$$\begin{array}{c} \sigma_{\nu_{t}}=2/3,\;C_{b1}=0.1355,\;C_{b2}=0.622,\;C_{\omega 1}=C_{b1}/\kappa^{2}+\left(1+C_{b1}\right)/\sigma_{\nu_{t}},\\ C_{\omega 2}=0.3,\;C_{\omega 3}=2,\;C_{\nu 1}=7.1,\;\kappa =0.41. \end{array}$$

Note that in contrast to the original Spalart–Allmaras RANS model, the trip term in Equation (11) is not considered in the present model formulation.

At the outer layer, the wall-adapting local eddy viscosity model (WALE) [32] is employed to solve the LES equations. In the WALE model, the subgrid-scale viscosity is expressed as [32]

(14) $$\nu_{t}={\left(C_{W}\Delta \right)}^{2}\frac{{\left(S_{ij}^{d}S_{ij}^{d}\right)}^{3/2}}{{\left({\bar{S}}_{ij}{\bar{S}}_{ij}\right)}^{5/2}+{\left(S_{ij}^{d}S_{ij}^{d}\right)}^{5/4}},$$

where $C_{W}=0.325$ is the model coefficient, $\Delta ={\left(\Delta_{x}\Delta_{y}\Delta_{z}\right)}^{1/3}$ the grid filter and $S_{ij}^{d}$ is the traceless symmetric part of the square of the velocity gradient tensor.

In the zonal LES approach, the resolved velocity and the pressure gradient from the LES calculation serve as boundary conditions for the inner-layer RANS simulation. Thereby, the wall stress from the RANS is returned as wall boundary condition for the LES calculation. Regarding the treatment of the thermal boundary layer, the turbulent thermal diffusivity $\alpha_{t}$ is calculated in the zonal LES approach based on Reynolds analogy assumptions as $\alpha_{t}=\nu_{t}/Pr_{t}$.

### 2.3. Improved Delayed Detached Eddy Simulation (IDDES)

Similar to [27], the Spalart–Allmaras eddy viscosity transport equation (see Equation (11)) is used in the present IDDES approach in order to achieve an eddy viscosity. Thereby, in contrast to the classical Spalart–Allmaras RANS model, a hybrid turbulent length-scale formulation, that blends between a RANS and a LES length scale, is used for the approximation of $\tilde{d}$ as [35]:

(15) $$\tilde{d}={\tilde{f}}_{d}\left(1+{\tilde{f}}_{e}\right)l_{RANS}+\left(1-{\tilde{f}}_{d}\right)l_{DES}.$$

Here, $l_{RANS}$ is a RANS-based turbulent length scale and $l_{DES}$ a length scale that depends on the grid width. The RANS-based turbulent length scale equals the distance to the wall $l_{RANS}=d$. The grid-based length scale is calculated as $l_{DES}=\Psi C_{DES}\Delta$, where Ψ is the low Reynolds number correction function (see [27]), $\Delta ={\left(\Delta_{x}\Delta_{y}\Delta_{z}\right)}^{1/3}$ the grid filter and $C_{DES}=0.65$ a model constant. The blending function ${\tilde{f}}_{d}$ in Equation (15) is defined in such a way that $l_{RANS}$ is predominantly used in regions with low mesh resolution and $l_{DES}$ in regions where the grid resolution is sufficient for LES. The elevation function $f_{e}$ aims at preventing an excessive reduction in the Reynolds stresses in the vicinity of the RANS–LES interface [35]. A detailed description of the IDDES model and the blending functions can be found in [27,35].

Similar to the zonal LES approach, the turbulent thermal diffusivity $\alpha_{t}$ is calculated in IDDES based on Reynolds analogy assumptions as $\alpha_{t}=\nu_{t}/Pr_{t}$.

## 3. Configuration and Numerical Procedure

**Figure 1:**
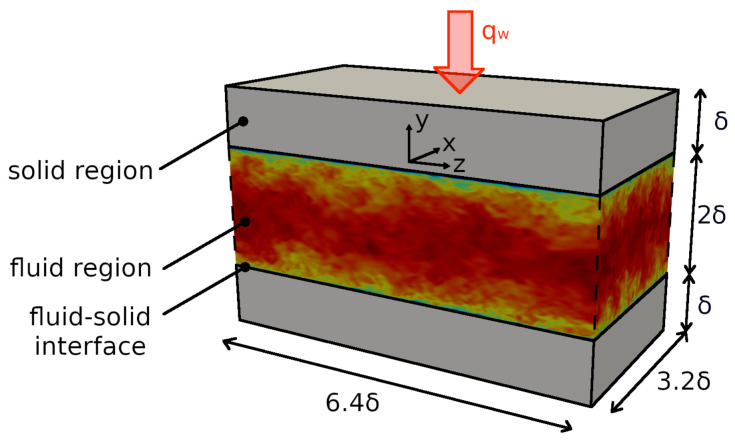
Channel flow configuration with conjugate heat transfer. Solid domains on top and bottom. Fluid region is located in the middle coupled via an interface with the solid domains.

In line with the numerical study of Flageul et al. [30], a turbulent heated channel flow test case with conjugate heat transfer was selected in this work. The heated channel flow was simulated for a fluid with a molecular Prandtl number of $Pr=\nu /\alpha^{f}=0.71$ and at Re${}_{\tau }$ = 395, 640, 1020, where Re${}_{\tau }=u_{\tau }\delta /\nu$ is the Reynolds number based on the friction velocity. A fluid-to-solid thermal diffusivity ratio of $G_{1}=\alpha^{f}/\alpha^{s}=1$ and a solid-to-fluid thermal conductivity ratio of $G_{2}=\lambda^{s}/\lambda^{f}=1$ were selected, leading to a thermal activity ratio of $K=1/G_{2}\sqrt{G_{1}}=1$[30]. These values of $G_{1}=G_{2}=K=1$ were selected in accordance with the reference DNS of [30] and represent the case of a coupled scalar with the same thermal properties in the fluid and solid region. A sketch of the computational domain used for the simulations is shown in Figure 1, where x, y and z are the spanwise, wall-normal and streamwise directions, respectively.

The entire computational domain has a length of 6.4δ, a width of 3.2δ and a height of 4δ, where δ is the channel half-height. Thereby, similar to [30], the fluid domain is bounded at −δ<y<δ and the solid domains are located at y>δ and y<−δ, respectively. Both solid domains have an height of δ, which ensures that the boundary condition used at the outer wall has no significant impact on the statistics at the fluid–solid interface [30].

Periodic boundary conditions were applied for the velocity and temperature in streamwise and spanwise directions. At the solid surface, a no-slip condition was employed for the velocity and a coupled thermal boundary condition was used for the temperature (see Equation (5)). The pressure and temperature gradients that drive the heat and fluid flow in the fluid region are adjusted dynamically to maintain a constant mass flux and mean mixed temperature. Therefore, the pressure and temperature were split into a periodic and a non-periodic part. Source terms for the non-periodic part, ${\bar{f}}_{x}^{U}$ and ${\bar{f}}^{T_{f}}$, were added to the momentum and temperature equation, respectively (see [34]).

Synthetic turbulence was used to initialize the channel flow simulations in the fluid region. Thereby, to avoid uncertainties caused by the initial solution, the start-up phase of the simulations was chosen to be long enough to ensure that the channel flow was fully developed before sampling started. A detailed description of the initialization method can be found in [36].

**Figure 2:**
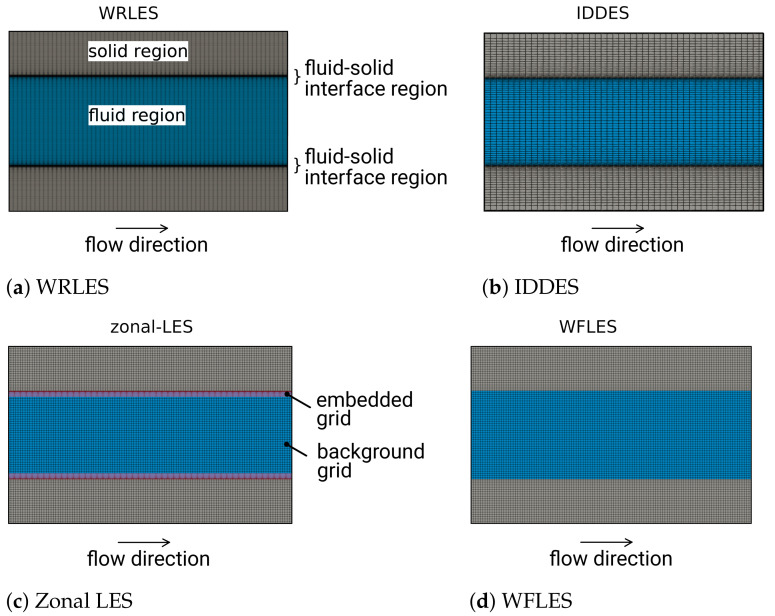
Numerical grids used in the (**a**) WRLES, (**b**) IDDES, (**c**) zonal LES and (**d**) WFLES approaches.

Three-dimensional block-structure numerical grids with different spatial resolutions were used for each Re${}_{\tau }$ and also for each LES near-wall modeling approach. To complete the DNS dataset, additional wall-resolved LES (WRLES) were conducted for each Re${}_{\tau }$, including the thermal cases for large Re-numbers for which DNS data were not available $\left(Re_{\tau }=640,1020\right)$. In the case of IDDES and WRLES, the numerical grid was refined towards the wall in order to ensure a non-dimensional wall distance $y_{w}^{+}$ smaller than one. A representation of each grid used for the WFLES, WRLES, IDDES and zonal LES approaches is shown in Figure 2. Thereby, the coarsest spatial resolutions for each of the test cases at $Re_{\tau }=395$ are shown (see Table 1 cases 1, 2, 5, 8).

The balance equations for turbulent flow with conjugate heat transfer were solved numerically using an incompressible version of *chtMultiRegionFoam* from the open-source software OpenFOAM v1912 [37]. Thereby, the temperature transport equation, the LES near-wall modeling approaches and the source terms that drive the channel flow were added to the source code. Regarding the fluid region, a merged PISO-SIMPLE ([38,39]) algorithm was employed for the pressure–velocity coupling. The solution procedure was applied with a second-order implicit backward-differencing scheme for the time integration, a low-dissipative second-order flux-limiting differencing scheme for the convection terms and a conservative scheme for the Laplacian and gradient terms. The time step of the simulations was chosen to be small enough to ensure that the Courant–Friedrichs–Lewy number remained smaller than one. Convergence optimization and acceleration techniques were incorporated in order to speed up the calculations. In particular, a geometric agglomerated algebraic multigrid solver was considered for the resolution of the pressure Poisson equation and for the momentum predictor. Convergence of the iterative procedure is obtained if the normalized residuals of all governing equations are reduced by more than three orders of magnitude within each time step.

Several wall-resolved and wall-modeled LESs have been carried out in the present work. A summary of all the test cases is given in Table 1.

## 4. Results

The comparative predictions of the conjugate heat transfer process due to conduction and convection as achieved by the different near-wall treatments for LES are divided into five parts. First, predictions of instantaneous temperature and velocity fields are presented and compared for the different modeling approaches. Then, fluid flow statistics from the different wall-modeled LES approaches are reported together with their effect on the heat transfer. The obtained predictions are compared with DNS and wall-resolved LES in order to analyze the accuracy of the different near-wall treatments. The comparison includes mean and rms values in the fluid and solid region as well as friction coefficients and Nusselt numbers. Next, results of a systematic grid-dependency study are presented that allow us to assess the influence of the spatial resolution on predictions obtained by the different near-wall treatments. Subsequently, the physical consistency of the modeling approach is investigated. In particular, predicted entropy generation rates obtained by the different wall-modeled LES approaches are compared with results of wall-resolved LES. Finally, the computational cost of the wall-modeled LES approaches is quantified and compared to classical wall-resolved LES methods in order to highlight the benefit of each near-wall treatment for practical LES.

### 4.1. Instantaneous Temperature and Velocity Fields

**Figure 3:**
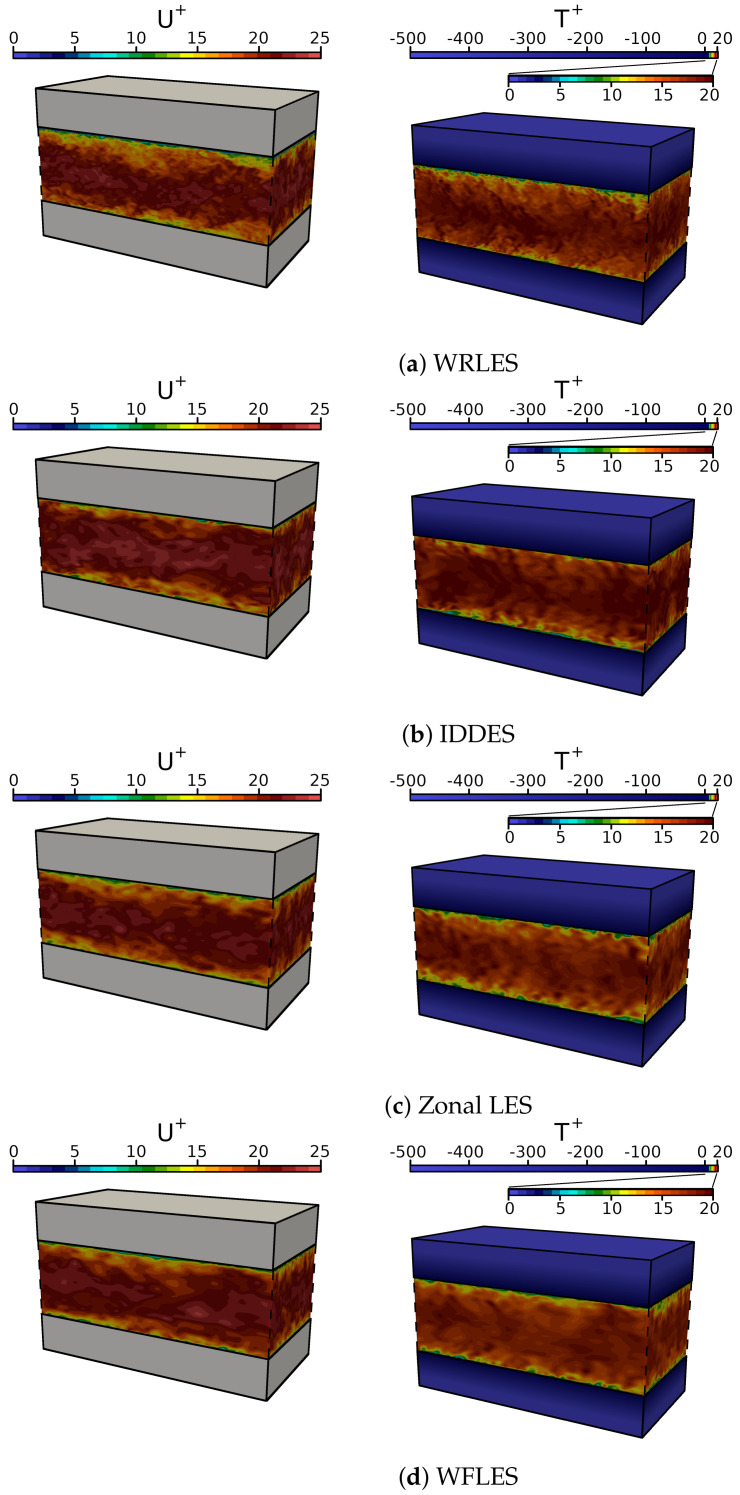
Snapshots of the instantaneous velocity field (**left**) and temperature field (**right**) predicted by means of (**a**) WRLES, (**b**) IDDES, (**c**) zonal LES and (**d**) WFLES. Results are shown for a channel flow with conjugate heat transfer at $Re_{\tau }=1020$ (see cases 21, 23, 26, 29 of Table 1).

Figure 3 presents predictions of instantaneous velocity and temperature fields in the channel flow configuration obtained by using (a) wall-resolved LES, (b) IDDES, (c) zonal LES and (d) LES with wall functions. Results are shown for the channel flow configuration at $Re_{\tau }=1020$ with medium spatial resolutions (cases 21, 23, 26, 29 of Table 1). Note that the temperature color scale is subdivided into a range for the fluid region and the solid region in order to better visualize the wide range of temperature scales.

It can be clearly seen in Figure 3 that the velocity and temperature fields are highly turbulent in the fluid region with steep velocity/temperature gradients close to the wall. Thereby, due to the finer grid resolution, more of the small-scale turbulent structures are resolved in the WRLES than in case of IDDES, zonal LES and WFLES. In contrast, the temperature field in the solid region is homogeneous distributed with a steep gradient in the wall-normal direction. Thereby, it appears that the predicted temperature fields obtained by the different wall-modeled approaches (IDDES, zonal LES, WFLES) are quite similar and compare well with the WRLES.

### 4.2. Fluid Flow Statistics and Impact on Heat Transfer

**Figure 4:**
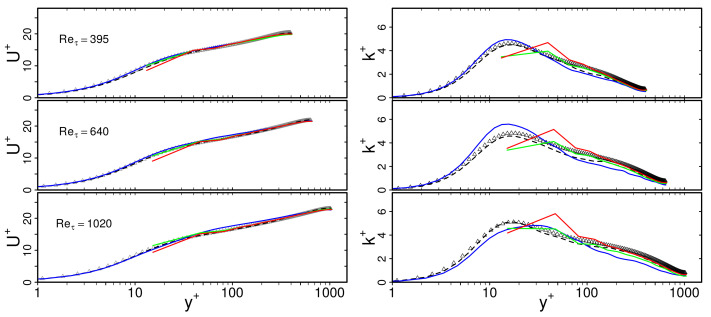
Dimensionless mean velocity $U^{+}$ and turbulent kinetic energy $k^{+}$ with respect to non-dimensional wall distance $y^{+}$ in turbulent heated channel flow at Re${}_{\tau }$ = 395, 640, 1020. Comparison of wall-modeled and wall-resolved LES (cases 1, 3, 6, 9, 11, 13, 16, 19, 21, 23, 26, 29 of Table 1) with DNS data of [40]. —: WFLES; —IDDES; —: zonal LES; - -: WRLES; Δ: DNS.

Figure 4 presents non-dimensional mean velocity $U^{+}$ and turbulent kinetic energy $k^{+}$ profiles as a function of dimensionless wall distance $y^{+}$ for the turbulent heated channel flow configuration at Re${}_{\tau }$ = 395, 640, 1020. Predictions of WFLES, IDDES and zonal LES are compared with DNS data from Abe et al. [40] as well as results of wall-resolved LES that have been computed in the present study (cases 1, 11, 21 of Table 1). Note that results of the wall-modeled LES are only shown for the medium spatial resolution (cases 3, 6, 9, 13, 16, 19, 23, 26, 29 of Table 1). Similar trends are found for the other spatial resolutions and are therefore not shown for the sake of clarity.

In the case of WRLES, it is apparent in Figure 4 that the agreement with DNS is very satisfactory for both $U^{+}$ and $k^{+}$. This confirmed the validity of the present WRLES results and allowed us to use this dataset as a reference for further assessment of the LES near-wall modeling strategies in this study. Regarding the predictions of the wall-modeled LES, it appears that mean velocity profiles agree very well with the DNS and WRLES data. This holds true for all near-wall treatments and also for all Re${}_{\tau }$ under consideration. Less good agreement can be observed for the predicted turbulent kinetic energy $k^{+}$. In particular, the peak value of $k^{+}$ is underestimated in the case of WFLES and zonal LES, while it is predominantly overestimated in the case of IDDES. Nevertheless, the overall agreement of the wall-modeled LES approaches is still satisfactory, which confirms the physical consistency of all LES near-wall modeling approaches under consideration in terms of turbulent channel flow.

**Figure 5:**
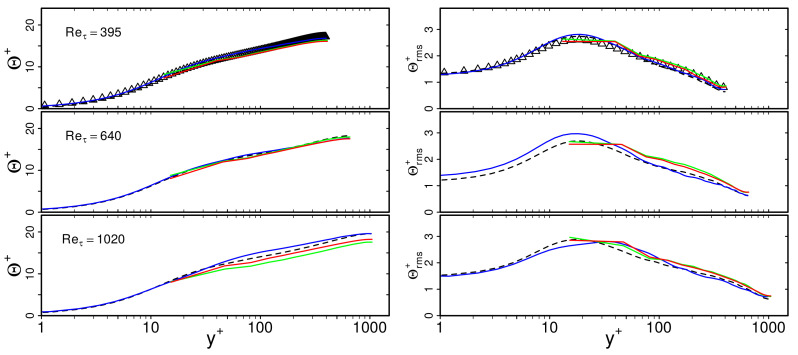
Dimensionless mean and rms temperature $\Theta^{+}$, $\Theta_{rms}^{+}$ as a function of non-dimensional wall distance $y^{+}$ for Re${}_{\tau }$ = 395, 640, 1020. Comparison of wall-modeled with wall-resolved LES (cases 1, 3, 6, 9, 11, 13, 16, 19, 21, 23, 26, 29). —: WFLES; —IDDES; —: zonal LES; - -: WRLES; Δ: DNS.

Figure 5 shows predicted non-dimensional mean temperature $\Theta^{+}$ and rms temperature $\Theta_{rms}^{+}$ profiles in the fluid region based on WFLES, IDDES and zonal LES treatment. Thereby, the non-dimensional temperature is defined as $\Theta^{+}=\left(T_{w}-T\right)/T_{\tau }$, where $T_{\tau }=q_{w}/\left(\rho^{f}c_{p}^{f}u_{\tau }\right)$ is the friction temperature with $q_{w}$ the wall heat flux which is defined in the case of wall-modeled LES as $q_{w}=\left(\alpha^{f}+\alpha_{t}\right){\left[\partial T/\partial y\right]}_{y=0}$. For comparison purposes, DNS data from [30] of a turbulent channel flow with conjugate heat transfer at Re${}_{\tau }=395$ were employed. For higher Re${}_{\tau }$, the results of wall-resolved LES (cases 1, 11, 21 of Table 1) were used for comparison because thermal statistics from DNS of this specific configuration at higher Re${}_{\tau }$ are not available in the literature. Note that in Figure 5, the value of $\Theta_{rms}$ is not zero at the wall. As indicated in [30], the temperature variance at the wall depends strongly on the value of the thermal activity ratio *K*. Thereby, lower values of *K* correspond to conjugate cases similar to isothermal boundary conditions, while higher values of *K* correspond to isoflux conditions at the thermal interface.

According to the fluid flow statistics in Figure 4, predictions of mean and rms temperature profiles based on WFLES, IDDES and zonal LES compare well with the WRLES reference data. Significant differences in the predictions from the individual wall-modeled LES approaches cannot be observed. Therefore, it can be concluded that WFLES, IDDES and zonal LES are able to reproduce thermal statistics in the fluid region properly in the case of turbulent channel flow with conjugate heat transfer. This holds true for all Re${}_{\tau }$ under consideration.

**Figure 6:**
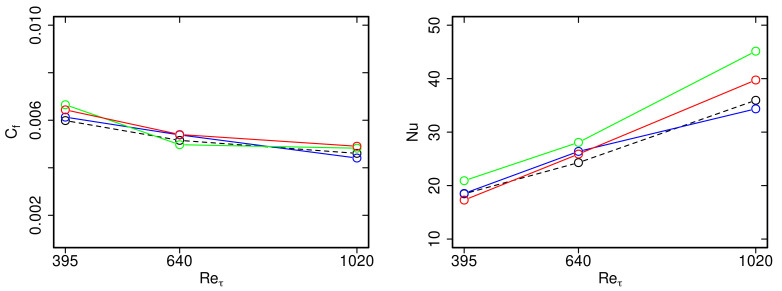
Skin friction coefficient $C_{f}$ und Nusselt number Nu as a function Re${}_{\tau }$. Comparison of wall-modeled LES with wall-resolved LES. —: WFLES; —IDDES; —: zonal LES; - -: WRLES.

Next, predictions of skin friction coefficients $C_{f}=2\tau_{w}/\left(\rho U_{b}^{2}\right)$ and Nusselt numbers $Nu=\left(\left(\alpha +\alpha_{t}\right)/\alpha \right)\cdot \delta {\left[\partial T/\partial y\right]}_{y=\delta }/\left(T_{w}-T_{\delta }\right)$ based on WFLES, IDDES and zonal LES are compared in Figure 6 with respect to WRLES for different Re${}_{\tau }$. Here, $\tau_{w}$ is the wall shear stress, $U_{b}$ the bulk velocity, $T_{w}$ the wall temperature and $T_{\delta }$ the temperature at y=δ. The integral quantities $C_{f}$ and Nu are particularly relevant for the development and design of industrial/engineering applications. Accurate predictions of such quantities are therefore important in the context of practical LES with near-wall modeling.

Regarding $C_{f}$, it can be clearly seen in Figure 6 that WFLES, IDDES and zonal LES are able to reproduce the skin friction coefficient accurately for all Re${}_{\tau }$ under consideration. In contrast, discrepancies in the prediction of Nusselt numbers are more significant. Thereby, in particular, the zonal LES approach slightly overestimates values of Nu, while results of WFLES and IDDES are still very close to the reference WRLES. Nevertheless, predictions of Nu obtained by zonal LES are still acceptable for wall-modeled LES, which leads to the conclusion that all wall-modeled LES approaches are suitable to predict heat and fluid flow statistics including skin friction coefficients and Nusselt number at the fluid region of turbulent channel flow with conjugate heat transfer.

**Figure 7:**
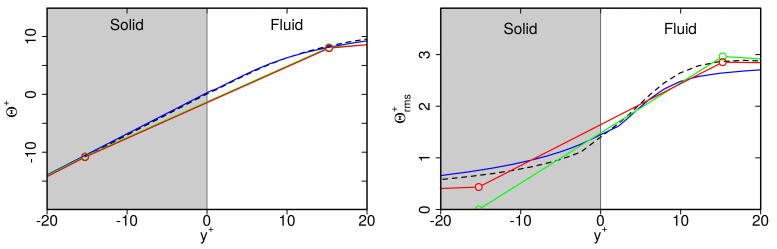
Dimensionless mean and rms temperature $\Theta^{+}$, $\Theta_{rms}$ as a function of non-dimensional wall distance $y^{+}$ for Re${}_{\tau }$ = 1020. Comparison of wall-modeled LES with wall-resolved LES. —: WFLES; —IDDES; —: zonal LES; - -: WRLES.

Finally, mean temperature $\Theta^{+}$ and rms temperature $\Theta_{rms}^{+}$ profiles in the solid region are presented in Figure 7. Predictions of WFLES, IDDES and zonal LES are compared with WRLES. Results are solely shown for the turbulent channel flow at Re${}_{\tau }$ = 1020. Similar trends are found for Re${}_{\tau }$ = 395 and 640 and are therefore not shown for the sake of clarity.

As can be clearly seen in Figure 7, predictions of $\Theta^{+}$ obtained by the individual wall-modeled LES approaches agree very well with the reference wall-resolved LES. This holds true for the interface as well as for the rest of the solid region. Similarly, profiles of $\Theta_{rms}^{+}$ are reproduced well by the wall-modeled LES, except in the case of zonal LES. Here, values of $\Theta_{rms}^{+}$ decrease too rapidly towards the outer wall. This unphysical behavior of the thermal fluctuation penetration may be caused by overestimated values of Nu at the thermal interface (see Figure 6), resulting in too intensive heat transfer in the context of zonal LES.

By examining predictions of heat and fluid flow statistics within the turbulent channel flow configuration with conjugate heat transfer, it turned out that WFLES, IDDES and zonal LES are able to reproduce the physics of such heated flows properly. The influence of the spatial resolution on the prediction accuracy is analyzed next.

### 4.3. Grid Dependency

**Figure 8:**
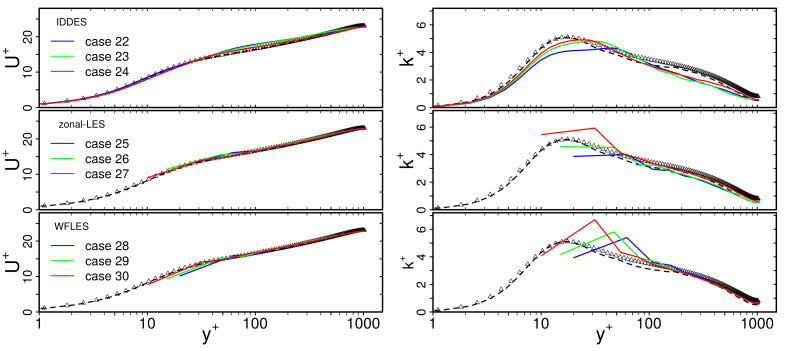
Dimensionless mean velocity $U^{+}$ and turbulent kinetic energy $k^{+}$ with respect to non-dimensional wall distance $y^{+}$ in turbulent heated channel flow at Re${}_{\tau }$ = 1020. Grid dependency study of wall-modeled LES (cases 2–10, 12–20, 22–20 of Table 1). Comparison with DNS data of [40] and wall-resolved LES (cases 1, 11, 21 of Table 1). —: coarse grid; —: medium grid; —: fine grid; - -: WRLES; Δ: DNS.

Figure 8 shows predictions of mean velocity $U^{+}$ and rms velocity $U_{rms}^{+}$ by IDDES, zonal LES and WFLES. Three different spatial resolutions are shown, denoted here as coarse, medium and fine. For comparison, DNS data of Abe et al. [40] and results of WRLES are depicted.

From Figure 8, it appears that predictions of $U^{+}$ are very close to the reference data and nearly independent of the spatial resolution. This holds true for IDDES, zonal LES and WFLES over the entire range of $y^{+}$. In contrast, predicted profiles of $k^{+}$ are more affected by the spatial resolution. In particular, the zonal LES and WFLES approaches predict a non-physical peak of $k^{+}$ close to the wall. Such a non-physical peak of $k^{+}$ might be caused by grid resolution requirements [41], the numerical schemes employed [42] and effects of the subgrid-scale model, among other numerical or modeling errors [43]. However, it can be seen that results are still reasonably close to the reference WRLES and DNS. Obviously, the grid dependency of mean and rms velocities obtained by wall-modeled LES is not very significant, at least for turbulent channel flow at $Re_{\tau }=1020$.

**Figure 9:**
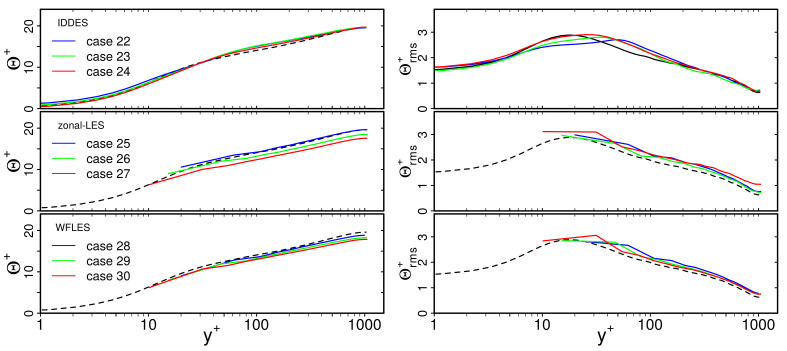
Grid-dependency of mean temperature $\Theta^{+}$ and rms temperature $\Theta_{rms}$ profiles obtained by WFLES, zonal LES, IDDES at Re${}_{\tau }$ = 1020. For legend, see Figure 8.

Next, the grid dependency of mean temperature $\Theta^{+}$ and rms temperature $\Theta_{rms}^{+}$ is analyzed in Figure 9.

Regarding mean temperature profiles, it is visible in Figure 9 that predictions based on IDDES compare very well with the reference DNS. This holds true for all spatial resolutions that are considered in the present study. In contrast, WFLES and zonal LES are more affected by the spatial resolution. Thereby, values of $\Theta^{+}$ are slightly underestimated, in particular for the fine grid resolution. As indicated in [41], such a log-layer mismatch might be caused by numerics, grid resolution requirements, effects of the subgrid model, etc. Regarding $\Theta_{rms}^{+}$, the influence of the spatial resolution is less significant. However, it can be observed that the peak value of $\Theta_{rms}^{+}$ is slightly shifted in the case of the IDDES approach and coarse grid. Nevertheless, similar to the fluid flow statistics, the grid dependency of mean and rms temperatures in the fluid region obtained by wall-modeled LES seems to be not very significant in the case of turbulent channel flow with conjugate heat transfer at $Re_{\tau }=1020$.

**Figure 10:**
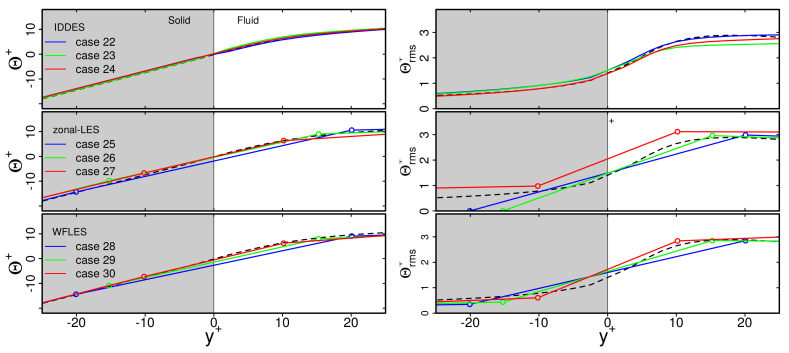
Predictions of mean temperature $\Theta^{+}$ and rms temperature $\Theta_{rms}$ profiles in the solid region of a turbulent channel flow with conjugate heat transfer at Re${}_{\tau }$ = 1020. Comparison of WFLES, zonal LES, IDDES with WRLES. For legend, see Figure 8.

Finally, the grid dependency of thermal statistics in the solid region were studied. For this purpose, Figure 10 presents mean temperature $\Theta^{+}$ and rms temperature $\Theta_{rms}^{+}$ profiles in the the vicinity of the wall. Results are shown for IDDES, zonal LES and WFLES for different spatial resolutions. For comparison, WRLES data were employed.

Just as is the case for the fluid region, predictions of mean temperature profiles are nearly independent of the spatial resolution in the solid region. Similarly, values of $\Theta_{rms}^{+}$ are more or less independent of the spatial resolution, except in the case of zonal LES. Here, $\Theta_{rms}^{+}$ decreases too rapidly towards the outer wall. This is particularly visible for the coarse and medium grid resolutions.

In summary, the grid dependency of heat and fluid flow statistics obtained by wall-modeled LES is not very significant. This holds true for IDDES, WFLES and zonal LES in the case of turbulent channel flow with conjugate heat transfer at $Re_{\tau }=1020$.

### 4.4. Physical Consistency of the Modeling

In addition to precise predictions of thermal and fluid flow statistics, it is also important that a wall-modeled LES approach is consistent with the second law of thermodynamics to ensure that thermodynamic processes, as they occur in heat and flow systems, are correctly described [7]. In the case of LES of turbulent fluid flows with conjugate heat transfer, the second law of thermodynamics can be written in the form of the filtered local entropy inequality at the continuum level [44,45,46,47] as:

(16) $$\frac{\partial \bar{\rho s}}{\partial t}+\frac{\partial }{\partial x_{j}}\left(\bar{\rho }\bar{U_{j}s}\right)+\frac{\partial }{x_{j}}\bar{\left(\frac{q_{j}}{T}\right)}={\bar{\Pi }}_{v}+{\bar{\Pi }}_{q}\geq 0,$$

where $\bar{\left(•\right)}$ denotes spatial filtering. The terms on the left-hand side are the local change, convection and flux of entropy density *s* [7]. The source terms on the right-hand side represent the local entropy production rates by viscous dissipation ${\bar{\Pi }}_{v}$ and by heat transfer ${\bar{\Pi }}_{q}$.

In the context of Navier–Stokes–Fourier fluid and LES with conjugate heat transfer, the mean of the filtered source terms, ${\bar{\Pi }}_{v}$ and ${\bar{\Pi }}_{q}$, can be formulated as [46,47]:

(17) $$\langle {\bar{\Pi }}_{v}\rangle =\underset{\langle {\bar{\Pi }}_{v}^{res}\rangle }{\underset{︸}{\langle \frac{\bar{\mu }}{\bar{T}}\left(\frac{\partial {\bar{U}}_{i}}{\partial x_{j}}+\frac{\partial {\bar{U}}_{j}}{\partial x_{i}}\right)\frac{\partial \bar{U_{i}}}{\partial x_{j}}\rangle }}+\underset{\langle {\bar{\Pi }}_{v}^{sgs}\rangle }{\underset{︸}{\frac{\langle \bar{\rho }\rangle }{\langle \bar{T}\rangle }\frac{{\langle \nu_{t}\rangle }^{3}}{\Delta^{4}C_{S}^{4}}}}$$

(18) $$\langle {\bar{\Pi }}_{q}\rangle =\underset{\langle {\bar{\Pi }}_{q}^{res}\rangle }{\underset{︸}{\langle \frac{\bar{\lambda }}{{\bar{T}}^{2}}\frac{\partial \bar{T}}{\partial x_{j}}\frac{\partial \bar{T}}{\partial x_{j}}\rangle }}+\underset{\langle {\bar{\Pi }}_{q}^{sgs}\rangle }{\underset{︸}{\frac{4\langle \bar{\rho }\rangle \langle {\bar{c}}_{p}\rangle \langle \nu_{t}\rangle }{3C_{OC}\pi^{4/3}C_{s}^{4/3}\langle Pr\rangle {\langle \bar{T}\rangle }^{2}}\langle \frac{\partial \bar{T}}{\partial x_{i}}\frac{\partial \bar{T}}{\partial x_{i}}\rangle }},$$

where 〈•〉 denotes temporal averaging, ${\left(•\right)}^{res}$ is the resolved contribution and ${\left(•\right)}^{sgs}$ represents the subgrid-scale contribution. In this work, $C_{OC}=1.34$ is the Obukhov–Corrsin constant [48] and $C_{S}$ the Smagorinsky coefficient [49]. The latter can be directly related to the WALE model as $C_{S}=C_{W}/\sqrt{11.27}$ [32].

**Figure 11:**
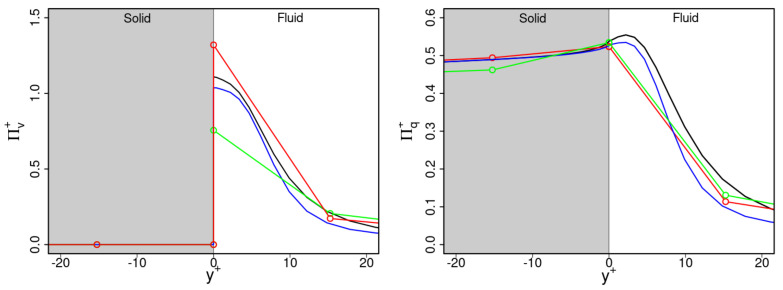
Predictions of entropy production rates by (**left**) viscous and turbulent dissipation and (**right**) heat transfer with mean and fluctuating temperature gradients in the vicinity of the wall. —: WRLES; —: IDDES; —: zonal LES; —: WFLES.

Figure 11 shows predicted entropy production rates (a) by viscous dissipation $\Pi_{v}$ and (b) by heat transfer $\Pi_{q}$ in the vicinity of the wall. Thereby, $\Pi_{v}$ is normalized as $\Pi_{v}^{+}=\Pi_{v}T_{w}\mu /\left(\rho^{2}u_{\tau }^{4}\right)$ and $\Pi_{q}$ is normalized as $\Pi_{q}^{+}=\Pi_{q}T_{w}^{2}\mu^{2}/\left(\rho^{2}\alpha u_{\tau }^{2}T_{\tau }^{2}\right)$, where $T_{w}$ is the temperature at the fluid–solid interface. Results are shown for the turbulent channel flow configuration at $Re_{\tau }=1020$ and medium grid resolution (cases 21, 23, 26, 29). Note that $\Pi_{v}^{+}$ is zero in the solid region due to the absence of velocity gradients.

It is visible in Figure 11 that $\Pi_{v}^{+}$ is zero inside the solid region, peaks at the solid–fluid interface and decreases rapidly with increasing wall distance within the fluid region. Obviously, irreversibilities in such flows occur predominantly at the solid–fluid interface where velocity gradients are high. A similar conclusion can be drawn for entropy generation by heat transfer $\Pi_{q}^{+}$, except inside the solid region, where steep temperature gradients lead to high entropy generation rates. Both characteristic trends are well captured by the different wall-modeled LES approaches, which confirms their physical consistency in terms of turbulent channel flow with conjugate heat transfer. It turns out that the behavior predictions close to the wall using zonal LES or WFLES are more affected by the dimensionless wall distance of the first cell at the solid surface than when using IDDES.

### 4.5. Computational Cost

**Figure 12:**
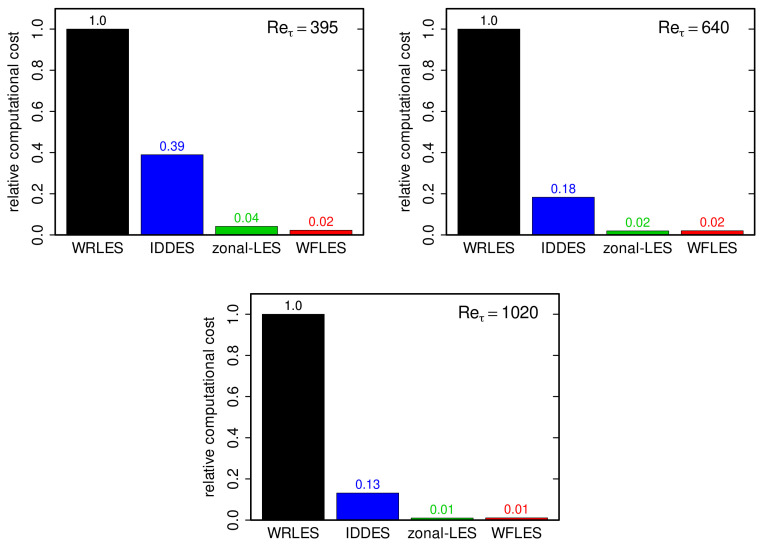
Relative computational costs of the different wall-modeled LES approaches with respect to the computational cost required for wall-resolved LES for turbulent channel flow with conjugate heat transfer at $Re_{\tau }=395,640,1020$.

One of the key objectives of using near-wall modeling in the context of LES is to reduce the computational effort in order to allow the calculation of high Reynolds number flows [19]. Therefore, it is of practical interest to address the required computational cost to achieve an acceptable prediction accuracy. For this purpose, Figure 12 shows the required relative computational cost of the different wall-modeled LES approaches with respect to wall-resolved LES. Thereby, the relative computational cost of a wall-modeled LES approach is defined in this work as the ratio of the CPU time spent for the calculation of a wall-modeled LES and the CPU time that is required for a wall-resolved LES of the same configuration. The computational cost was estimated in the present study on a Linux 3.10.0-514.26.1.el7.x86 64 Red Hat 4.8.5-11 (x86 64) system using an Intel(R) Core(TM) i5-6600K CPU @ 3.50GHz and 32GiB RAM. Only one CPU core was used to quantify the computational cost and the maximal Courant–Friedrichs–Lewy number of the simulations was set to CFL=0.3. The use of only one CPU core for estimating the runtime performance was chosen because the parallel scalability is not in the scope of this study. Note that the relative computational cost depends generally not only on the near-wall treatment model, but also on the selected test case, the particular code implementation and the solution procedure applied.

Regarding the turbulent channel flow test case with conjugate heat transfer, it was found in this work that the computational cost of WFLES and zonal LES is about 50–100 times lower than in the case of WRLES. The computational cost of IDDES is significantly higher than that of WFLES and zonal LES but still 3–10 lower compared to WRLES. Furthermore, it can be observed that the relative computational cost of all tested wall-modeled LES approaches decreases with increasing Reynolds number. However, due to the considerable extra work to generate numerical grids in the case of IDDES and especially for zonal LES, this work suggests that WFLES offers, in particular, a promising near-wall modeling strategy for LES of conjugate heat transfer in realistic engineering applications.

## 5. Conclusions

The impact of different wall-modeled LES approaches, namely, LES with non-equilibrium wall functions (WFLES), two-layer RANS–LES (zonal LES) and improved delayed detached eddy simulation (IDDES), on the heat transfer predictions has been investigated in a turbulent channel flow with conjugate heat transfer at $Re_{\tau }=395,640,1020$. Thereby, the heat transfer process involved the interaction of conduction within the channel solid wall and convection from the solid wall surface by the fluid flow. In addition, the physical consistency, accuracy and computational cost of the different near-wall modeling strategies have been assessed.

Using available DNS data as a reference, the fluid flow statistics obtained were first evaluated and compared to wall-resolved LES (WRLES). Then, the effect of these predictions on the heat transfer was assessed. The following important findings are worth mentioning:

(i) WFLES, IDDES and zonal LES are able to reproduce the physics of turbulent channel flow with conjugate heat transfer properly.
(ii) The grid dependency of fluid flow statistics obtained by IDDES, WFLES and zonal LES is not very significant. The same is valid for the thermal field.
(iii) The computational cost of IDDES, WFLES and zonal LES of turbulent channel flow with conjugate heat transfer is considerably lower than in the case of WRLES. In particular, WFLES and zonal LES allow accurate predictions with a reasonable computational cost.
(iv) The relative computational cost of the wall-modeled LES decreases with increasing Reynolds number.

Finally, it was found that IDDES and especially zonal LES require considerable extra work to generate numerical grids. Therefore, in the authors’ opinion, WFLES also offers an especially promising near-wall modeling approach for LES of conjugate heat transfer in realistic engineering applications. An entropy generation analysis using the various models was carried out. From this analysis, it turned out that the viscous entropy production is zero inside the solid region due to the prevailing zero velocity gradient, peaks at the solid–fluid interface and decreases rapidly with increasing wall distance within the fluid region. Except inside the solid region, where steep temperature gradients lead to high (thermal) entropy generation rates, a similar conclusion can be drawn for the entropy generation by the heat transfer process. 

## Figures and Tables

**Table 1:** Summary of the evaluation study for LES with near-wall modeling. ($x^{+},y^{+},z^{+}$): dimensionless distance in x-, y-, z-direction; $y_{w}^{+}$: dimensionless wall distance of the first cell at the solid surface; WRLES: wall-resolved LES; IDDES: improved delayed detached eddy simulation; zonal LES: two-layer RANS–LES approach; WFLES: LES with non-equilibrium wall functions.

Case	($x^{+},y^{+},z^{+}$)	$y_{w}^{+}$	Cells Solid	Cells Fluid	Reτ	Rebulk	Wall Treatment
1	(10.5,9.5,21.07)	0.25	2,131,200	2,131,200	395	13,773	WRLES
2	(19.7,14.2,39.5)	0.25	606,208	606,208	395	13,773	IDDES
3	(15.8,13.6,31.6)	0.25	947,200	947,200	395	13,773	IDDES
4	(13.2,12.2,26.4)	0.25	1,363,968	1,363,968	395	13,773	IDDES
5	(19.7,39.5,39.5)	19.8	81,920	81,920	395	13,773	zonal LES
6	(15.8,26.3,31.6)	13.2	192,000	192,000	395	13,773	zonal LES
7	(13.2,19.8,26.4)	9.9	368,640	368,640	395	13,773	zonal LES
8	(19.7,39.5,39.5)	19.8	81,920	81,920	395	13,773	WFLES
9	(15.8,26.3,31.6)	13.2	192,000	192,000	395	13,773	WFLES
10	(13.2,19.8,26.4)	9.9	368,640	368,640	395	13,773	WFLES
11	(8.5,9.2,17.1)	0.32	9,100,800	9,100,800	640	23,834	WRLES
12	(25.6,14.8,51.2)	0.32	1,011,200	1,011,200	640	23,834	IDDES
13	(17.1,14.2,34.2)	0.32	2,275,200	2,275,200	640	23,834	IDDES
14	(12.8,13.6,25.6)	0.32	4,044,800	4,044,800	640	23,834	IDDES
15	(25.6,40,51.2)	20	204,800	204,800	640	23,834	zonal LES
16	(17.1,32,34.2)	16	576,000	576,000	640	23,834	zonal LES
17	(12.8,21.2,25.6)	10.6	864,000	864,000	640	23,834	zonal LES
18	(25.6,40,51.2)	20	204,800	204,800	640	23,834	WFLES
19	(17.1,32,34.2)	16	576,000	576,000	640	23,834	WFLES
20	(12.8,21.2,25.6)	10.6	864,000	864,000	640	23,834	WFLES
21	(10.2,13.9,20.4)	0.4	16,179,200	16,179,200	1020	40,478	WRLES
22	(27.2,16.5,54.4)	0.5	2,275,200	2,275,200	1020	40,478	IDDES
23	(20.4,15.4,40.8)	0.5	4,044,800	4,044,800	1020	40,478	IDDES
24	(16.3,14.2,32.6)	0.5	6,320,000	6,320,000	1020	40,478	IDDES
25	(27.2,52.3,54.4)	20.1	576,000	576,000	1020	40,478	zonal LES
26	(27.2,49.4,54.4)	15.3	633,600	633,600	1020	40,478	zonal LES
27	(27.2,48.8,54.4)	10.2	691,200	691,200	1020	40,478	zonal LES
28	(19.7,52.3,39.5)	20.1	576,000	576,000	1020	40,478	WFLES
29	(27.2,49.4,54.4)	15.3	633,600	633,600	1020	40,478	WFLES
30	(27.2,48.8,54.4)	10.2	691,200	691,200	1020	40,478	WFLES

## Data Availability

The data presented in this study are available on request from the corresponding author.

## Abbreviations

The following abbreviations are used in this manuscript:

LES	Large eddy simulation
DNS	Direct numerical simulation
RANS	Reynolds-averaged Navier–Stokes
CFD	Computational fluid dynamics
WRLES	Wall-resolved LES
WFLES	LES with wall functions
Zonal LES	Two-layer RANS–LES
IDDES	Improved delayed detached eddy simulation
